# Effects of X-Ray Irradiation on Biological Parameters and Induced Sterility of *Ephestia elutella*: Establishing the Optimum Irradiation Dose and Stage

**DOI:** 10.3389/fphys.2022.895882

**Published:** 2022-05-03

**Authors:** Jun Zhao, Shujun Li, Lu Xu, Chengjun Li, Qi Li, Youssef Dewer, Kongming Wu

**Affiliations:** ^1^ Key Laboratory for Green Prevention and Control of Tobacco Diseases and Pests in Huanghuai Growing Area, Institute of Tobacco Research, Henan Academy of Agricultural Sciences, Zhengzhou, China; ^2^ Key Lab of Food Quality and Safety of Jiangsu Province-State Key Laboratory Breeding Base, Institute of Plant Protection, Jiangsu Academy of Agricultural Sciences, Nanjing, China; ^3^ Henan Branch of China National Tobacco Corporation, Zhengzhou, China; ^4^ Phytotoxicity Research Department, Central Agricultural Pesticide Laboratory, Agricultural Research Center, Giza, Egypt; ^5^ State Key Laboratory for Biology of Plant Diseases and Insect Pests, Institute of Plant Protection, Chinese Academy of Agricultural Sciences, Beijing, China

**Keywords:** *Ephestia elutella*, sterile insect technique, X-ray irradiation, induced sterility, male mating competitiveness

## Abstract

The sterile insect technique (SIT) is widely used for the inundative release of sterile mass-reared males to control lepidopteran pests. SIT based on X-ray irradiation is an eco-friendly alternative to chemical control. However, its use in *Ephestia elutella*, a stored tobacco pest currently controlled with insecticides, is poorly explored. This study aims to investigate the effects of X-ray irradiation on *E. elutella* to determine the optimal sterilizing dose and processing developmental stage for improving SIT application. The pupal stage was most suitable for irradiation that was more tolerant than the other insect stages including eggs, larvae, and adults. Subsequently, male pupae were irradiated with X-ray doses of 0, 50, 100, 150, 200, 250, and 300 Gy and mated with unirradiated females. Their emergence, longevity, egg number, egg hatch rate, developmental duration, survival rate, induced sterility, and male mating competitiveness were evaluated. The results suggest that a dose of 200 Gy can be applied to effectively induce sterility in male pupae, after which induced sterility and male mating competitiveness can be balanced by increasing the release ratio (sterile:normal). When the release ratio was 15:1, it was found that 71.91% of the wild population could be suppressed. The results of this study show that the SIT based on X-ray irradiation can be successfully used to manage *E. elutella,* improves our understanding of the biological effects of the SIT, and expands its future application to the control of other pests.

## 1 Introduction

The tobacco moth, *Ephestia elutella* (Hübner) (Lepidoptera: Pyralidae), is a polyphagous pest infesting stored products such as tobacco, nuts, dried fruits, and cereals (Baltaci et al., 2009). Its larvae are economically important as they mainly feed on stored products, creating holes in the packaging and resulting in serious economic losses. The larvae of *E. elutella* directly eat the tobacco leaf mesophyll in tobacco barns, decreasing the rate of tobacco filamentation and the quality of tobacco leaves (Ashworth, 1993). Together with *Lasioderma serricorne,* they cause an annual loss of approximately 1% of stored tobacco worldwide (Trematerra, 2020). Multiple approaches have been developed and applied to control *E. elutella*, but current control practices depend primarily on insecticides (Rumbos et al., 2018). However, the resistance of these organisms to chemical insecticides has limited the control of stored-product pests, and excessive use of insecticides has created negative environmental problems (Hubert et al., 2008; Ou et al., 2021). Alternative eco-friendly approaches are warranted to reduce dependence on insecticides for the sustainable management of *E. elutella*.

Some of the eco-friendly methods applied to control *E. elutella* include insecticide (alpha-cypermethrin)-coated net protection, pheromone trapping, controlled atmosphere treatments, physical control with higher and lower temperatures, and biological control using natural enemies (Wang et al., 2021). However, integrated pest management (IPM), which involves the combination of different pest control methods and the various techniques used for biological and cultural/mechanical control, is highly needed to prevent pests from causing extensive damage. The IPM can reduce pesticide use because the control techniques will only be used when the insect densities exceed the economic injury level (Trematerra, 2012). Ionizing radiation is considered an alternative technology in IPM to control lepidopteran pests (Yun et al., 2016), there is an urgent need to develop sterile insect techniques (SITs) based on ionizing radiation for *E. elutella*.

SITs are eco-friendly species-specific control method used in pest management. Sterile insects are not self-replicating and cannot establish wild populations in the environment, which reduces the adverse environmental impact of this method (Mastrangelo et al., 2010; Du et al., 2019). Released sterile insects mate with the wild ones in the target area and cause reproductive failure, reducing infestation levels in the offspring. SITs involve mass rearing, sterilization, and release methods that produce competitive sterile males for mating with wild females (Krüger et al., 2018). Exposure to ionizing radiation is a common treatment method for inducing sterile insects in SITs (Mastrangelo et al., 2018). In the late 1970s and the early 1980s, preliminary studies investigated the effect of SITs on *E. elutella* control through gamma radiation, showing population reduction or elimination by released sub-sterilized males in targeted areas (Brower, 1979; Brower, 1982), but there are few later studies on the sterilizing effects of ionizing radiation. Gamma-emitting sources from radioactive cobalt-60 or cesium-137 are typically used for irradiation in SITs. However, obtaining radioactive materials has become increasingly difficult because of government control, radioisotope suppliers going out of business, and increasing restrictions on radioisotopes in response to the threat of international terrorism. The X-ray irradiation process is a safer alternative to gamma radiation, easily obtained and operated, and can fulfill the requirements of SIT programs (Yamada et al., 2014), but the effects of X-ray radiation on *E. elutella* control have not yet been fully understood.

X-ray irradiation-based SITs have been used to treat a variety of pest infestations because of its several advantages, including discontinuous radiation emission, no radioactive waste, low transport costs, easy operation, and excellent penetration depth (Yun et al., 2016). The X-ray radiation dose used in the SIT varies for different insect species. Large doses of X-rays made *L. serricorne* incapable of reproduction (Runner, 1916), whereas a dose of 150 Gy of X-ray was sufficient to prevent the production of adults from pupae in *Omphisa anastomosalis* (Follett, 2006) and egg hatch from the progeny of irradiated neonates, larvae, and pupae of *Amorbia emigratella* (Follett, 2008). *Epiphyas postvittana* required a 400 Gy X-ray dose for sterilization (Follettt and Snook, 2012), but a 100% sterility could be induced using a lower dose of 40 Gy radiation in *Aedes albopictus* (Yamada et al., 2014). The optimal sterilizing dose and processing stage for application vary among insect species, and the optimal dose is a crucial initial step in the SIT to achieve high sterility without a lethal effect and mate dysfunction in insect species. Previously, Brower (1979) determined 50% (220 and 350 Gy) and 99% (450 and 550 Gy) sterility doses for females and males, respectively, through gamma radiation in *E. elutella*, suggesting that gamma radiation-based SIT to control this moth is feasible. However, no studies on the optimal irradiation dose of X-rays to control *E. elutella* have been conducted. To evaluate the optimal sterilizing dose for insects, irradiation-affected biological features should be assessed using various parameters, such as emergence, longevity, number of eggs, hatch rate, and mortality (Lanouette et al., 2017). Therefore, the effective dose at different developmental stages and the biological quality of irradiated *E. elutella* using X-rays need to be investigated.

The objective of this study is to investigate the optimal sterilizing dose and developmental stage for X-ray irradiation on *E. elutella* by evaluating the mortality, emergence, number of eggs, hatch rate, longevity, developmental duration, survival rate, induced sterility, and male mating competitiveness. The results will facilitate the application of SITs based on X-ray radiation to control *E. elutella* by setting sterility parameters. Additionally, the findings can provide some basic knowledge about the effectiveness of X-ray radiation on the quarantine treatment of *E. elutella*.

## 2 Materials and Methods

### 2.1 Insect Culture

The *E. elutella* colony originated in a tobacco warehouse in Xuchang City, Henan Province, China. They were maintained successively under insectarium conditions at the Institute of Tobacco Research, Henan Academy of Agricultural Sciences, at 27 ± 1°C, relative humidity 75 ± 5%, and a photoperiod of 16 h light:8 h dark. The hatched larvae were fed a previously described artificial diet (Wang et al., 2021), and fan-shaped papers were provided to promote pupation. The pupae were collected in petri dishes (diameter, 9 cm) and kept in plastic insect tanks (diameter, 15 cm; height, 25 cm) that were sealed at the bottom with stainless steel gauze. The emerged adults were allowed to lay eggs on filter papers under stainless steel gauze and were provided 10% a sugar solution as a nutrition source for oviposition. The artificial diets were periodically replaced by new ones.

### 2.2 X-Ray Irradiation Procedure

**FIGURE 1:**
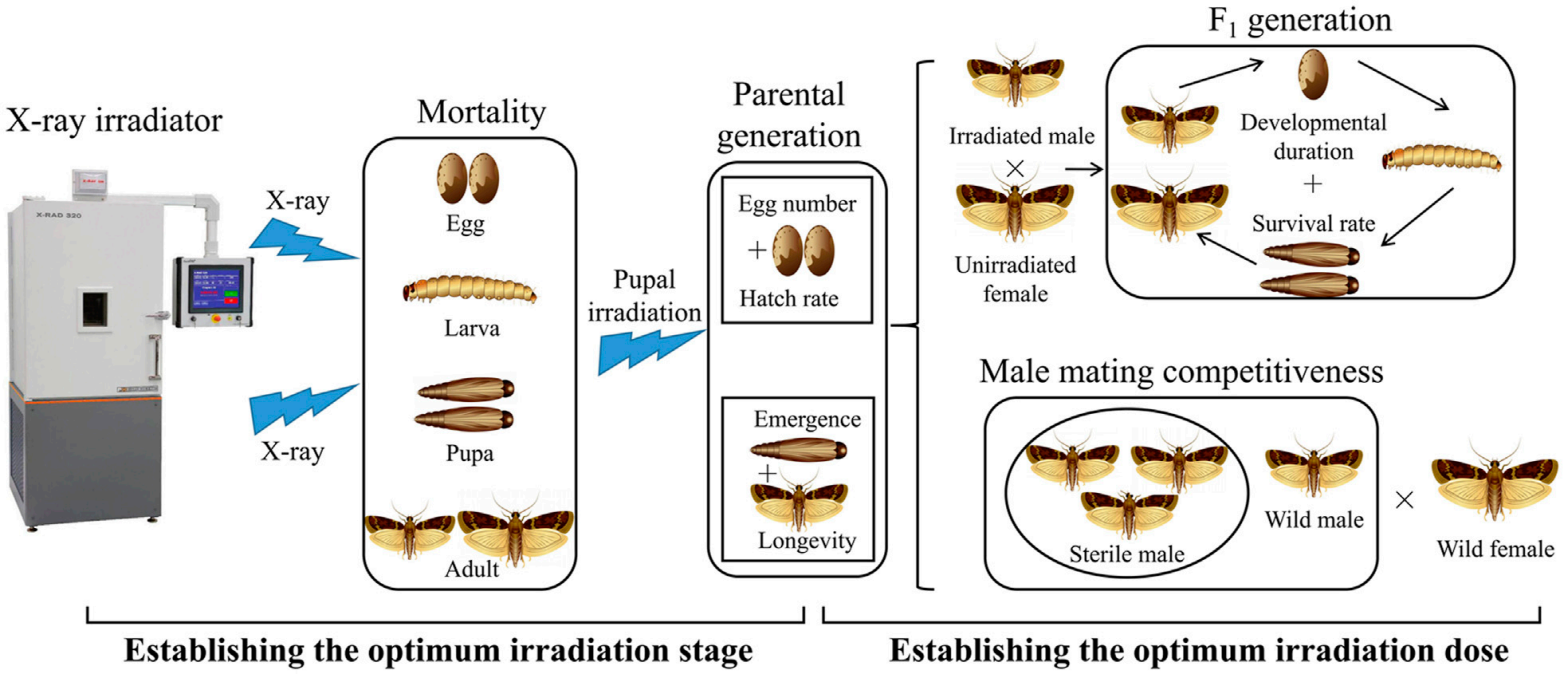
Flowchart showing the methodology used for this study.

X-ray irradiation was applied using a commercially available X-RAD 320 irradiator (F2 beam hardening filter 1.5 mm Al, 0.25 mm Cu, 0.75 mm Sn; Precision X-ray Inc., Branford, CT, United States) at ambient temperature, operated at 320.00 kV and 12.50 mA at a rate of 1.45 Gy/min at the irradiation position. This facility was calibrated annually to ensure its integrity and compliance with nationally recognized standards. The actual dose was within ±2% of the target dose. Irradiation samples were placed at the center of the lead stage in the irradiator system to receive X-rays. X-rays were emitted from the X-ray tube above the stage. The irradiation voltages, currents, and times were automatically determined according to the set irradiation doses in the facility control panel. The target irradiation doses were set at 0 (no irradiation), 50 (2076 s), 100 (4,135 s), 150 (6,212 s), 200 (8,281 s), 250 (10,338 s), and 300 Gy (12,415 s). When the cumulative dose was reached, the irradiator was stopped and the samples were removed. During the study, the measured dosage rate was 1.44–1.47 Gy/min. The experiment design process is summarized in Figure 1.

### 2.3 Effects of Irradiation at Different Developmental Stages on Mortality

The three-day-old eggs laid on filter paper were irradiated using X-rays. The treatment was replicated three times, and each replicate contained at least 30 eggs, including the controls. Next, the irradiated eggs were transferred to petri dishes, counted, and provided an artificial diet. The mortality of eggs (non-incubation of larvae) was checked under a microscope daily after the first egg hatched. The 3rd–4th instar larvae were placed in plastic cups and exposed to X-rays. Each replicate contained 50 larvae, and the treated and control groups were repeated three times. The irradiated larvae were introduced into plastic insect tanks with an artificial diet. After 72 h, the number of dead larvae was determined by observing any detectable movement after touching the larvae with a brush. The mortality rate of each group was calculated as the ratio of the number of dead larvae to the total number of larvae. The seven-day-old pupae (72 h prior to adult emergence and sex ratio = 1:1) were collected on petri dishes and exposed to X-ray irradiation. Each group with 30 pupae was treated in triplicate. The irradiated pupae were then transferred to plastic insect tanks for emergence, and the percentage of unemerged pupae was calculated to determine the mortality rate. Before irradiation, the adults (two days old) were anesthetized using CO_2_ and transferred to plastic containers. Groups of 30 adults (sex ratio = 1:1) were collected, irradiated, and then placed into the plastic insect tanks with 10% a sugar solution. Survivors were evaluated after 72 h. Three replicates were used for each group, including controls. The eggs, larvae, pupae, and adults of *E. elutella* were subjected to X-ray radiation at doses of 0 (control), 50, 100, 150, 200, 250, and 300 Gy and then reared under the same conditions.

### 2.4 Effects of Pupal Irradiation on Emergence, Longevity, Egg Number, and Hatch Rate

Groups of 60 male pupae 72 h before emergence were introduced into petri dishes (diameter, 90 mm) and irradiated at 50, 100, 150, 200, 250, and 300 Gy, whereas the control pupae were not irradiated. All experiments were conducted in triplicate for each dose and the control. The irradiated pupae were transferred to plastic insect tanks and maintained there until they emerged. The pupae that did not emerge normally were counted to evaluate emergence rates. The emerged male adults from irradiated pupae and non-irradiated virgin adult females were anesthetized, paired, and transferred to each tank for mating and 10% a sugar solution feeding. Three replicates of 30 pairs were used for each treatment, including the controls. Filter papers were placed under the stainless-steel gauze of tanks for oviposition and replaced every 48 h. The set of tanks was kept under the same insectarium conditions until all adults died. The longevity of the male adults was determined by recording the daily mortalities in each tank. The number of eggs that failed to hatch was counted to evaluate the effect of radiation treatment on induced sterility.

### 2.5 Effects of Pupal Irradiation on Developmental Duration and Survival Rate

Based on the aforementioned experimental, the male pupae exposed to 200 Gy were chosen to analyze the effects of irradiation on the developmental duration and survival rate of the F1 generation. Groups of 60 male pupae, collected 72 h before emergence, were irradiated at 200 Gy. The irradiated pupae were transferred to plastic insect tanks and subsequently maintained under the same insectarium conditions. After emergence, 30 pairs [at a sex ratio of 1:1 (male: female)] of IM × NF (I = irradiated; N = normal, unirradiated; M = male; F = female) were allowed to mate and held in each tank, which was provided 10% a sugar solution. Filter papers were used to collect the eggs, which were checked daily. The hatched number and developmental duration of the F_1_ eggs were recorded daily after the first egg hatched. The hatch rate was considered the egg survival rate. Thirty newly hatched larvae were transferred to plastic insect tanks with an artificial diet, and the developmental duration and survival rate of F_1_ larvae were calculated when the larvae pupated. Groups of 30 pupae each were maintained until emergence. The developmental duration of F_1_ pupae was calculated when the first pupa emerged. The number of pupae that emerged from the total number of pupae was calculated as the survival rate. All experiments were conducted in triplicate for each dose and the control.

### 2.6 Effects of Irradiation on Male Mating Competitiveness

Based on the two pupal irradiation experiments, the male pupae were exposed to 200 Gy to examine the effects of irradiation on male mating competitiveness. First, 72 h before emergence, the male pupae were collected, irradiated, and transferred to clean plastic insect tanks for emergence and 10% a sugar solution feeding. Then, a total of 0, 30, 90, 180, 270, 360, and 450 irradiated males were moved to clean cages containing 30, 30, 30, 30, 30, and 0 non-irradiated normal females. The release ratios (irradiated male:normal male; IM/NM) were 0:1 (normal control group), 1:1 (competition group), 3:1 (competition group), 6:1 (competition group), 9:1 (competition group), 12:1 (competition group), 15:1 (competition group), and 1:0 (sterile control group). Thirty females (non-irradiated virgins, 1–2 days old) were introduced into each cage for mating competition. Eggs were collected using a filter paper and checked daily. The number of eggs hatched was recorded daily after the first egg hatched to calculate the hatch rates. There were three replicates for each control group (sterile and normal) and competition group (0:1, 1:0, 1:1, 3:1, 6:1, 9:1, 12:1, and 15:1). Male mating competitiveness was evaluated based on the hatch rates.

### 2.7 Statistical Analysis

Mortality, egg hatch rates, pupal emergence rates, survival rate, and induced sterility were arcsine-transformed to achieve a normal distribution. One-way analysis of variance and Tukey’s HSD test (*p* < 0.05) were performed to test for significant differences between the controls and X-ray irradiation treatments. Survival rates between groups were compared using Kaplan–Meier survival analysis. Effective dose (ED) values for mortality at different developmental stages were estimated using Probit analysis. All data are presented as the mean ± SD. Induced sterility and male mating competitiveness index were calculated as follows: (Du et al., 2019)

$$IS\left(induced\;sterility\right)=\left[\left(1-Hs\right)-\frac{Hs}{Hn}\right]\times 100\%$$

$$C\left(male\;mating\;competitiveness\;index\right)=\frac{Hs-Hc}{Hn-Hs}\times \frac{N}{S}$$

Where Hs = hatch rate of the sterile control group, Hc = hatch rate of the competition group, Hn = hatch rate of the normal control group, N = number of normal males, and S = number of sterile males.

## 3 Results

### 3.1 Mortality

**FIGURE 2:**
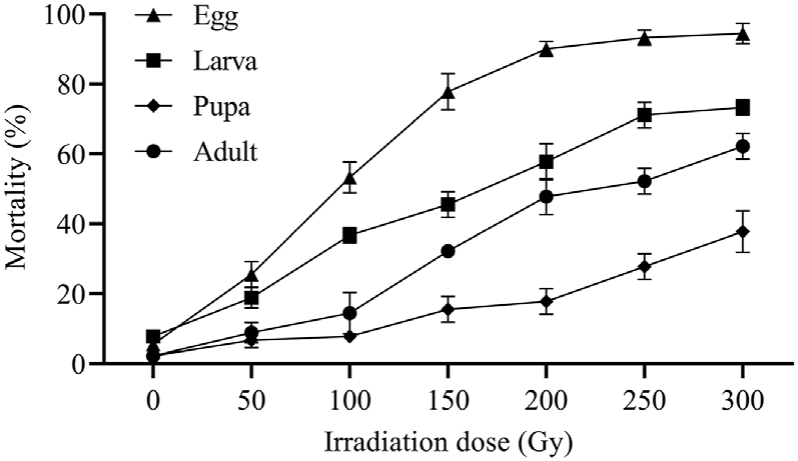
Mortality of egg, larva, pupa, and adult of *E. elutella* irradiated at X-ray doses of 50, 100, 150, 200, 250, and 300 Gy.

The mortality rates of eggs, larvae, pupae, and adults of *E. elutella* after exposure to different irradiation doses are shown in Figure 2. Egg mortality rates at doses ≥50 Gy were significantly higher than those at 0 Gy (F_1,4_ = 40.50; *p* = 0.0031). The egg mortality significantly increased from 25.56% to 93.33% with increasing radiation dose, and most mortality rates reached 94.44% at 300 Gy (F_5,12_ = 108.56; *p* = 0.0000). No significant differences in egg mortality (90.00%, 93.33%, and 94.44%) were observed among those irradiated at 200, 250, and 300 Gy.

Larval mortality at 0 Gy were significantly lower than those at doses of 50 Gy and above (F_1,4_ = 20.00; *p* = 0.0111). Larval mortality differed significantly from 50 to 300 Gy (F_5,12_ = 58.17; *p* = 0.0000). A mortality rate of 73.33% was observed at 300 Gy, which was not statistically different from that at 250 Gy (71.11%).

Irradiation doses ≥50 Gy had significant effects on pupal mortality compared to those at 0 Gy (F_6,14_ = 20.14; *p* = 0.0000). No statistical difference in pupal mortality was observed between radiation doses of 50 and 100 Gy. The mortality rate was significant at 150 Gy and continued to increase by 37.78% at 300 Gy (F_3,8_ = 8.43; *p* = 0.0074). There was no significant difference in pupal mortality (15.56% vs. 17.78%) between 150 and 200 Gy.

Adult mortality at doses ≥100 Gy was significantly higher than those at doses ≤50 Gy (F_1,4_ = 16.00; *p* = 0.0161). Irradiation doses ranging from 100 to 300 Gy caused a significant increase in adult mortality (from 14.44% to 62.22%) (F_4,10_ = 46.84; *p* = 0.0000). Adults irradiated with 50 and 0 Gy radiation did not show significant mortality rates. Adult mortality rates were similar between 200 and 250 Gy.

The ED_50_ and ED_99_ values for different developmental stages were determined based on the mortality data (Table 1). The ED_50_ values for the mortality of eggs, larvae, pupae, and adults were 96.16, 165.83, 380.17, and 229.61 Gy, respectively. The ED_99_ values for the mortality of eggs, larvae, pupae, and adults were 558.23, 2033.04, 3,867.62, and 2,294.76 Gy, respectively. These results showed that *E. elutella* eggs were most susceptible to X-ray irradiation, whereas pupae were the most tolerant.

**TABLE 1:** Effective dose values of different developmental stages of *Ephestia elutella* based on mortality.

Stage	Number	Slope (SE)	ED_50_ (95%CI) (Gy)	ED_99_ (95%CI) (Gy)	χ^2^ (*df*)	*p* values
Egg	545	3.05 (0.25)	96.16 (85.27–106.47)	558.23 (448.96–750.17)	0.900 (4)	0.925
Larva	542	2.14 (0.24)	165.83 (146.89–188.11)	2033.04 (1,275.50–4,157.75)	5.244 (4)	0.263
Pupa	536	2.31 (0.32)	380.17 (314.92–514.51)	3,867.62 (1997.54–12,283.69)	0.605 (4)	0.963
Adult	534	2.33 (0.26)	229.61 (203.70–266.53)	2,294.76 (1,421.66–4,824.10)	3.509 (4)	0.477

### 3.2 Emergence, Longevity, Egg Number, and Hatch Rate

**FIGURE 3:**
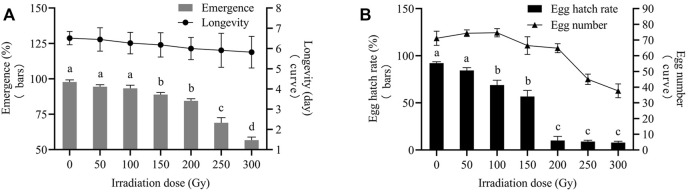
Biological reactions of X-ray-irradiated *E. elutella* male pupae. Effects of pupal irradiation on emergence and longevity **(A)**. Effects of pupal irradiation on egg number and egg hatch rate **(B)**. Error bars represent the SD from the mean of three independent replicates. Different letters indicate significant differences among treatments as determined by one-way ANOVA followed by Tukey’s HSD test (*p*-value < 0.05)

Irradiation of male pupae at doses ≥150 Gy significantly reduced emergence compared to the control (0 Gy). Furthermore, 84.44%, 68.89%, and 56.67% of irradiated male pupae developed into male adults at 200, 250, and 300 Gy, respectively, the differences were statistically significant (F_2,6_ = 42.82; *p* = 0.0003). Male pupa emergence irradiated at 50 and 100 Gy was not different. Male longevities at all irradiation doses were not significantly different from those of the controls. The average longevity of males in the controls was 6.51 days. The average longevities ranged from 5.45 to 5.82 days for males derived from pupae, which were exposed to radiation doses of 50–300 Gy (Figure 3A).

After irradiation at 50 and 100 Gy, the average number of eggs laid by females was 74.33 and 74.67, respectively (not a statistically significant difference). When exposed to doses ≥150 Gy, the number of eggs significantly decreased compared to the control. The number of eggs began to decrease significantly at 150 Gy and continued to decrease at 250 Gy. There was no significant difference in the number of eggs (66.50 and 64.83) between the 150 and 200 Gy doses. At 300 Gy, the number of eggs decreased to 37.67, which was statistically significant at 250 Gy (F_1,58_ = 36.94; *p* = 0.0000). The egg hatch rate tended to decrease with increasing doses. The egg hatch rate at 50 Gy was 84.44%, which was not significantly different from that of the control. The egg hatch rate reduced sharply to 10.00% at doses above 200 Gy versus that at 100 and 150 Gy. The egg hatch rates of 8.89% and 7.78% were lower at 250 and 300 Gy than that at 200 Gy; nevertheless, the difference among the three was not statistically significant (Figure 3B).

### 3.3 Developmental Duration and Survival Rate

**FIGURE 4:**
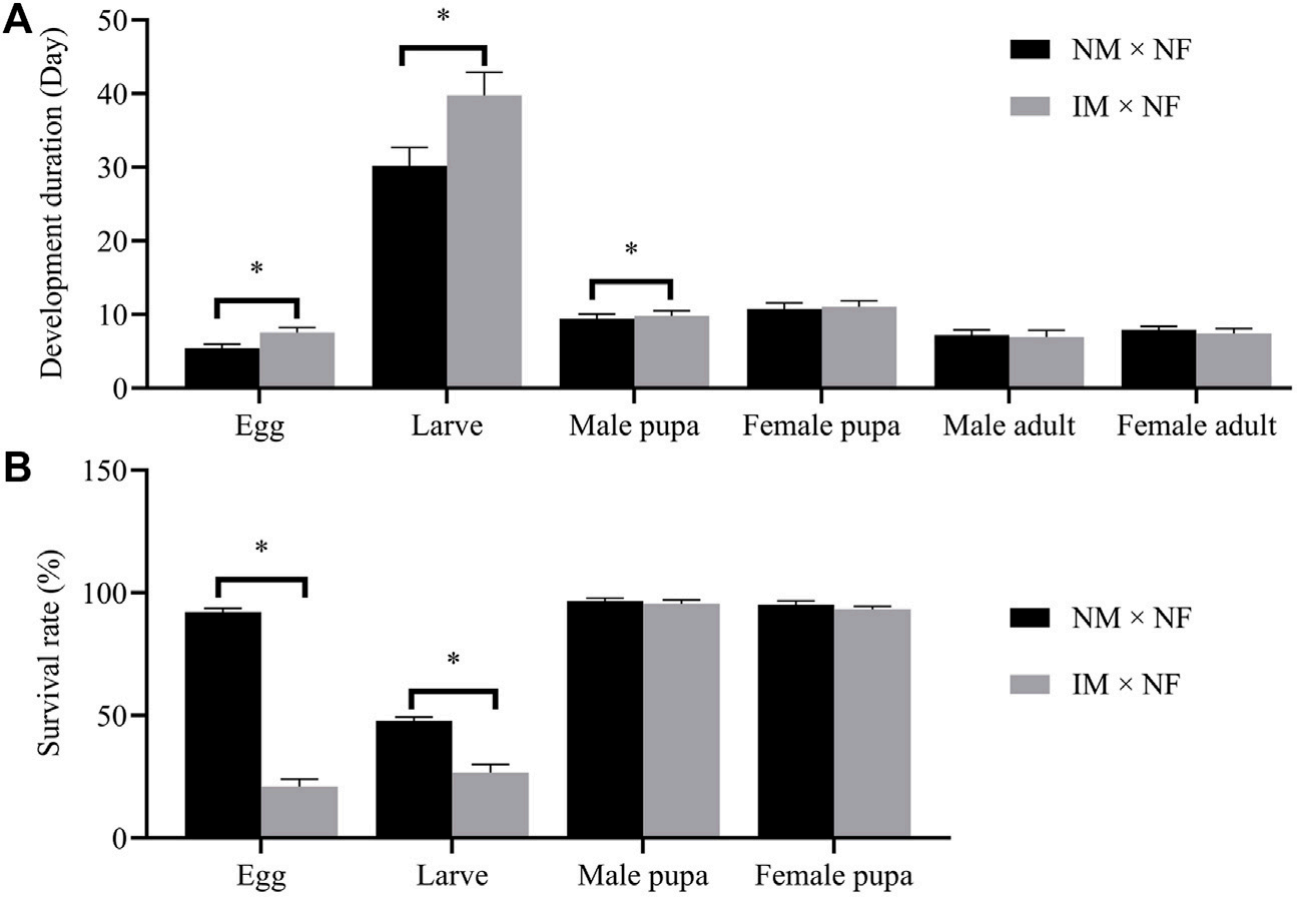
Developmental duration **(A)** and survival rate **(B)** of F_1_ developmental stages laid in the cross IM × NF compared to in the control cross NM × NF. Error bars represent the SD from the mean of three independent replicates. Asterisks indicate significant differences with *p*-value < 0.05 compared to the control group.

The developmental duration of F_1_ eggs, larvae, pupae, and adults following X-ray irradiation of male pupae is shown in Figure 4A. A dose of 200 Gy significantly prolonged the development of F_1_ eggs and larvae. The developmental durations of F_1_ eggs (7.58 days) and larvae (39.75 days) were longer than those of the controls (5.42 and 30.17 days), whereas those of F_1_ pupae and adults were not affected by this radiation dose, which was not statistically significant compared to the control. The developmental durations of F_1_ male pupa and female pupa were 9.83 and 11.08 days, respectively, and those of the F_1_ male adult and female adult were 6.92 and 7.42 days, respectively. These differences were not statistically significant.

No relationship was observed between the radiation dose of 200 Gy and the survival rate of F_1_ pupae. However, this radiation dose significantly decreased the survival rates of F_1_ eggs and larvae. After the irradiation of male pupae at 200 Gy, the survival rates of F_1_ eggs and larvae dropped from 92.22% and 47.78% to 21.11% and 26.67%, respectively. The survival rates of F_1_ male pupa and female pupa were 95.56% and 93.33%, respectively, which were not statistically significant compared to the control (Figure 4B).

### 3.4 Male Mating Competitiveness

**FIGURE 5:**
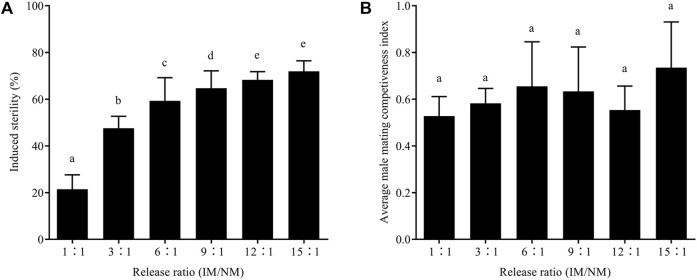
Effects of irradiation on induced sterility **(A)** and male mating competitiveness index **(B)** of *E. elutella* at different releasing ratios. Error bars represent the SD from the mean of three independent replicates. Different letters indicate significant differences among treatments (ANOVA and Tukey’s HSD test, *p* < 0.05)

After mating with males irradiated as male pupae, the average hatch rate of eggs laid by normal females decreased with the increasing release ratio, and the differences were significant; however, the average number of eggs was not significantly affected by the release ratio (Table 2). The average induced sterility increased significantly with the release ratio; the induced sterility scores from the release ratios of 1:1, 3:1, 6:1, 9:1, 12:1, and 15:1 were 21.46 ± 4.50%, 47.59 ± 3.60%, 59.30 ± 8.11%, 64.71 ± 6.31%, 68.31 ± 2.70%, and 71.91 ± 3.15%, respectively (Figure 5A).

**TABLE 2:** Number of eggs and hatch rates of *Ephestia elutella* at different releasing ratios.

Types of Irradiation	Releasing ratio (IM:NM)	Replicates	Egg number	Egg hatch rate (%)
Pupal irradiation	0∶1	3	77.25 ± 2.22 a	92.50 ± 2.92 a
	1∶1	3	75.25 ± 3.30 a	64.17 ± 2.50 b
	3∶1	3	76.00 ± 2.94 a	40.00 ± 3.33 c
	6∶1	3	63.50 ± 9.57 b	29.17 ± 4.17 d
	9∶1	3	65.50 ± 3.87 b	24.17 ± 2.92 de
	12∶1	3	66.75 ± 3.30 b	20.83 ± 2.50 e
	15∶1	3	65.25 ± 4.43 b	17.50 ± 2.92 e
	1∶0	3	62.75 ± 4.35 b	9.17 ± 1.25 f

Different letters indicate significant differences among treatments (ANOVA and Tukey’s HSD test, *p* < 0.05).

There was no significant difference in the male mating competitiveness index between the release ratio groups. After male pupal irradiation, the average C values were 0.54 ± 0.03 after male pupal irradiation. Therefore, assuming the male mating competitiveness of irradiated males was equal to that of control males, 1.7–2.0 irradiated males were needed to achieve the same induced sterility. Furthermore, C was independent of the release ratio of the pupal irradiation (Figure 5B).

## 4 Discussion

X-ray irradiation was applied in *E. elutella* to determine the feasibility and effectiveness of SIT by testing the biological response to the radiation dose. SITs require an optimum irradiation dose that aims to induce effective sterility with minimal impact on overall biological quality because insects may not be adequately sterile at low doses and are less competitive at high doses. First, the mortality rates of eggs, larvae, pupae, and adults were compared at different radiation doses. The mortality rates of pupa were lowest among those during the other insect stages, suggesting that radio tolerance varies with insect stages. The values of ED_50_ and ED_99_ reflect the radio-sensitivities of different developmental stages in the increasing order of eggs. The pupae were most tolerant to X-ray radiation which was determined as the optimum radiation insect stage, due to the reduction of rapidly dividing cells, making a less susceptible stage to radiation damage. The optimum dose that balanced the sterility of the insect with its vigor could be determined from these doses because the endpoint of the ionizing radiation in the SIT was not mortality but the prevention of further biological development and reproduction of the target pests (Hallman, 2011). The irradiation doses used ranging from 50 to 300 Gy did not cause 100% mortality in four insect stages, indicated that the optimal radiation dose was in this range. Furthermore, pupae were exposed to these radiation doses after evaluating biological parameters for a specific radiation dose to induce sterility. The advantage of irradiation at the pupal stage is that it is more convenient and pupae are easier to transport and handle than fragile eggs, active larvae, and adults, as observed in studies on *A. albopictus* (Oliva et al., 2013) and *Bactrocera dorsalis* (Chang et al., 2015).

Adult emergence rate is an important quality parameter for measuring pupal irradiation (Mastrangelo et al., 2010). The rate of male adult emergence was dose-dependent. The male adult emergence values increased with the increment of radiation doses. This suggested that higher radiation doses caused morphological damage to male adults *E. elutella.* Similar studies have reported a significant decline in male adult emergence after the pupal irradiation of *Helicoverpa assulta* at ≥100 Gy (Park et al., 2017). Male adult longevity of *E. elutella* was not influenced by radiation doses in the range of 50–300 Gy compared to the controls (no irradiation); this is consistent with studies on *Drosophila suzukii* (Krüger et al., 2018) and *Helicoverpa armigera* (Kim et al., 2015). It can be speculated that somatic cells are not damaged by radiation doses. Adult longevity may reflect the overall fitness of males and be strongly linked to the release of sterile male (Yamada et al., 2014). Our results indicated that unaffected adult male longevity ensured sufficient competitiveness with the wild males in targeted areas.

Egg number and hatch rate are important quality parameters for evaluating sterility in SITs. Male pupal irradiation significantly reduced the number of eggs and the hatch rate, suggesting that X-ray irradiation caused the destruction of reproductive cells and further induced sterility. Similar findings were reported for *A. albopictus* (Balestrino et al., 2010)*.* Irradiation treatment of other insects, such as *H. armigera* (Park et al., 2017) and *Cydia pomonella* (Botto and Glaz, 2010), did not affect the egg number of females but significantly reduced the hatch rate. These differences suggest insect species specificity for irradiation. The egg hatch rate decreased significantly following irradiation at 200, 250, and 300 Gy. However, the male adult emergence and egg number at 200 Gy was not significantly reduced when compared to those at 250 and 300 Gy, thus indicating that a dose of 200 Gy could be applied to induce sterility.

X-ray radiation at 200 Gy were not affected developmental durations of F_1_ pupae and adults. Similar results have been reported in developmental duration studies on *H. armigera* (Kim et al., 2015) and *Amyelois transitella* (Light et al., 2015), where the pupal duration was not changed by X-ray radiation. This implies that a dose of 200 Gy could stimulate defenses involved in cell repair mechanisms. Our study showed that the survival rates at pupae stages of the F_1_ generation were not affected after exposure to 200 Gy, and survival rates of F_1_ eggs and larvae significantly decreased compared to the control, indicating that F_1_ eggs and larvae are more sensitive to X-ray radiation than pupae. However, the survival rates of F_1_ larvae in the control group were below 50% compared to other insect stages, for which the higher natural mortality response was included. Therefore, natural mortality reduction should be considered in future studies. For the success of SIT, it is important that irradiated insects destined for field release can survive during the period of mating activity; the survival rates in this study were similar to those observed in *Ceratitis capitata* and *Anastrepha fraterculus* after exposure to X-ray radiation (Mastrangelo et al., 2010). The transgenerational effects of pest control programs that integrate the SITs on developmental duration and survival rate further confirmed the optimal radiation dose of 200 Gy applied to male pupae. In addition, male pupal irradiation solved the problem of separating males and females to prevent females from entering the released sterile male populations.

Mating competitiveness is a fundamental parameter in SIT. Sterilized males must mate as effectively as wild males so that they can induce sterility in the target pest population. The measure of sterility typically employed in irradiation studies of lepidopteran pests involves documentation of the egg hatch rate for treated moths and subsequent tracking of the offspring through the next generation to determine inherited sterility (Haff et al., 2020). Therefore, we determined the mating competitiveness index and induced sterility in the SIT laboratory trial of *E. elutella.* The average male mating competitiveness index was 0.54 after irradiation at 200 Gy, which was not affected by the release ratio. However, the induced sterility of male *E. elutella* bred with irradiated pupae increased with the increase of release ratio. This finding could be due to the reduction of egg hatch rate. At the release ratio of 15:1, induced sterility reached 71.91%. Although the male mating competitiveness index was low in our study, this deficiency can be compensated by increasing the release ratio. The wild *C. capitata* population was successfully suppressed by increasing the release ratio of sterile males to 100:1 (Rendón et al., 2004). Our data suggested that *E. elutella* populations were completely suppressed or eliminated by the release of sterile males at ratios over 15:1. These results agreed with those of Brower (1979) and Mastrangelo et al. (2018), where increased release ratios were necessary for the suppression/eradication of *E. elutella* and *A. fraterculus* populations.

Nonetheless, SIT programs should balance sterility and mating competitiveness to maximize induced sterility in wild populations (Parker and Mehta, 2007). Our experiments on the effects of X-ray irradiation showed that 200 Gy applied to male pupae induced sterility without any adverse effects on biological quality, indicating considerable competitiveness with wild males of the targeted population. X-ray irradiation at 250 Gy for the control of *E. elutella* infestation should be further tested in a large-scale targeted area to achieve application acceptance. Therefore, future research should determine the feasibility and effectiveness of X-ray irradiation-based SIT against field *E. elutella* populations. Based on the results of this study, X-ray irradiation fulfills the application requirements of SIT programs because it shows high effectiveness, predictability, and reproducibility in the sterilization of male moths.

In summary, this study is the first to demonstrate the effects of X-ray irradiation-based sterile insect techniques on *E. elutella.* The male pupa was determined as a suitable processing stage to study the effects of X-ray irradiation by evaluating the tolerance of *E. elutella* to radiation at each developmental stage. An X-ray dose of 200 Gy is optimal as an effectively induced sterility dose for pupal irradiation. The relationship between induced sterility and male mating competitiveness was balanced by increasing the release ratios. The findings presented herein showed that SIT based on X-ray irradiation is a scientific and feasible method that can be used to control *E. elutella*, and it is recommended as an alternative to chemical insecticides and a phytosanitary quarantine treatment*.*

## Data Availability

The original contributions presented in the study are included in the article/Supplementary Material, further inquiries can be directed to the corresponding authors.
